# Evidenced‐based approach facilitates choosing between different topical compounds in acne vulgaris

**DOI:** 10.1111/jdv.20590

**Published:** 2025-03-25

**Authors:** Georgios Nikolakis, Christos C. Zouboulis

**Affiliations:** ^1^ Departments of Dermatology, Venereology, Allergology and Immunology, Staedtisches Klinikum Dessau Brandenburg Medical School Theodor Fontane and Faculty of Health Sciences Brandenburg Dessau Germany

Acne vulgaris is the most common skin disease, and its pathogenesis has been the subject of extensive research over the last 3 to 4 decades. Its most important pathophysiologic pillars include a dysfunction in the keratinization of the follicular epithelium, which is tightly intertwined with an aberrant immunologic response, both innate and adaptive.^1^ The interplay between epithelial cells and commensal bacteria such as *Cutibacterium acnes* leads to a proinflammatory microenvironment. Other factors, such as seborrhea, altered fatty acid composition and hormonal imbalance also contribute to the pathogenesis of this complex disease.^2^

Several topical compounds have been developed and are effectively used for the treatment of mild‐to‐moderate acne, targeting one or more of the aforementioned factors. Retinoids exhibit both antiproliferative, comedolytic (keratolytic) and anti‐inflammatory effects. Benzoyl peroxide (BPO) has bactericidal properties against *C. acnes* as well as mild comedolytic and anti‐inflammatory properties. As for antibiotics, topical clindamycin is particularly effective against inflammatory acne lesions, but it can also affect biofilm and follicular microcomedone formation.^3^ Erythromycin is also an effective alternative, but concerns have been raised regarding its potential to easily promote *C. acnes* resistance.

In a recent meta‐analysis by Kakpovbia et al.^4^ different topical treatments were compared by evaluating randomized control trials for topical treatments in acne with focus on efficacy. Publications based on absolute inflammatory and non‐inflammatory lesion counts and the semiquantitative method of assessing treatment success based on the Investigator's Global assessment scale were included. Despite the dual targets of several compounds, the authors did not notice any significant differences among adapalene, azelaic acid, BPO, dapsone, erythromycin, clindamycin, salicylic acid, tretinoin and tazarotene.

Combination treatments with retinoid or topical antibiotic with BPO were the most effective among all treatments for inflammatory acne, while for non‐inflammatory acne combination treatments with BPO and clindamycin did not outperform BPO^4^: The authors provide evidence supporting a differentiation of the therapeutic approach in patients according to their acne phenotype in order to achieve the maximal efficacy and contribute in avoiding unnecessary prescription of topical clindamycin for chronic skin disorders, which raises antibiotic resistance concerns.^5^ The compound is ranked 125th most commonly prescribed medicine in the US and remains on the World Health Organization list of Essential Medicines, while its use is very often not limited to “in label” indications. Dermatologists frequently face constructive criticism concerning antibiotic stewardship practices. Despite this, they are among the few specialties that are familiar with the anti‐inflammatory properties of antibiotics and they prescribe them effectively in sub‐bactericidal doses for inflammatory skin diseases, such as acne vulgaris, rosacea and hidradenitis suppurativa.^6^

Another important factor that plays a crucial role in topical treatments is the tolerability profile. Compounds which cause irritation, erythema, dryness, burning or stinging on a regular basis are more likely to be associated with a reduced compliance. This is particularly important in the real‐world situation, where patients receive a treatment from their dermatologist and are seen in three‐month intervals, without the exhausting compliance practices which usually follow a randomized controlled study. The authors found that BPO alone or in combination with clindamycin is better tolerated than certain retinoids combined with BPO.

The extensive and rigorous inclusion criteria used did not allow for the inclusion and efficacy evaluation of new compounds such as the topical antiandrogen clascoterone and the new selective retinoid trifaroten. Despite this, this systematic review and metanalysis provide the evidence of an indirect comparison between commonly prescribed single agents and combination treatments and provides the rationale for a correct and phenotype‐oriented therapeutic strategy.

## FUNDING INFORMATION

None.

## CONFLICT OF INTEREST STATEMENT

Georgios Nikolakis has received honoraria and travel grants from UCB, Novartis, Almirall, BMS, Abbvie and Elli Lilly, and his institution received honoraria from Mölnlycke GmbH for his participation in advisory boards. Christos C. Zouboulis has received disease‐relevant consulting/lecture honoraria from Estée Lauder, L'Oréal, NAOS‐BIODERMA and PPM. His departments have received grants from his participation as a clinical and research investigator for AstraZeneca, Boehringer Ingelheim, BMS, Brandenburg Medical School Theodor Fontane, EADV, European Union, German Federal Ministry of Education and Research, GSK, Incyte, InflaRx, MSD, Novartis, Relaxera, Sanofi and UCB. He is chair of the ARHS Task Force group of the EADV and Editor of the EADV News.

## ETHICAL APPROVAL

Not applicable.

## ETHICS STATEMENT

Not applicable.

## Data Availability

Data sharing is not applicable to this article as no new data were created or analysed in this study.
